# Subtype-Independent Dysregulation of the Notch Signaling Pathway and Its miRNA Regulators in Breast Cancer

**DOI:** 10.3390/biomedicines13123065

**Published:** 2025-12-12

**Authors:** Elżbieta Mitka-Krysiak, Katarzyna Król-Jatręga, Piotr Ossowski, Nikola Zmarzły, Krzysztof Bereza, Paweł Ordon, Wojciech Kulej, Tomasz Sirek, Agata Sirek, Kacper Boroń, Maciej Boroń, Dariusz Boroń, Beniamin Oskar Grabarek

**Affiliations:** 1 Collegium Medicum, WSB University, 41-300 Dabrowa Gornicza, Poland; katarzynakroljatrenga@gmail.com (K.K.-J.); drpiotrossowski@gmail.com (P.O.); nikola.zmarzly@wsb.edu.pl (N.Z.); wojciechkulej@hotmail.com (W.K.); drtskierka@gmail.com (T.S.); agatasirek06@gmail.com (A.S.); boronmaciej7b@gmail.com (M.B.); dariusz@boron.pl (D.B.); beniamin.grabarek@wsb.edu.pl (B.O.G.); 2 Department of Mother and Child Health, Faculty of Health Sciences, Institute of Nursing and Midwifery, Jagiellonian University Medical College, 31-008 Kraków, Poland; krzysztof.bereza@uj.edu.pl; 3 Department of Plastic Surgery, Faculty of Medicine, Academia of Silesia, 40-555 Katowice, Poland; q375@icloud.com; 4 Department of Plastic and Reconstructive Surgery, Hospital for Minimally Invasive and Reconstructive Surgery in Bielsko-Biała, 43-316 Bielsko-Biala, Poland; 5 Faculty of Medicine and Health Sciences, Andrzej Frycz Modrzewski University in Kraków, 30-705 Kraków, Poland; 6 Department of Gynecology and Obstetrics, TOMMED Specjalisci od Zdrowia, 40-662 Katowice, Poland; 7 Department of Gynecology and Obstetrics with Gynecologic Oncology, Ludwik Rydygier Memorial Specialized Hospital, 31-826 Kraków, Poland

**Keywords:** breast cancer, Notch signaling pathway, microRNA, molecular marker

## Abstract

**Background/Objectives**: The Notch signaling pathway regulates cell fate, proliferation, and differentiation, and its dysregulation has been implicated in various cancers, including breast cancer. MicroRNAs (miRNAs) are critical post-transcriptional regulators that can modulate Notch pathway components. The aim of this study was to identify miRNAs that may potentially regulate the expression of Notch pathway-related genes across five molecular subtypes of breast cancer in Polish women. **Methods**: Tumor and adjacent normal tissue samples were collected from 405 patients with five breast cancer subtypes: luminal A (*n* = 130), HER2-negative luminal B (*n* = 100), HER2-positive luminal B (*n* = 96), non-luminal HER2-positive (*n* = 36), and triple-negative breast cancer (*n* = 43). Gene expression was profiled using mRNA microarrays and validated with RT-qPCR and ELISA. Candidate regulatory miRNAs were identified by miRNA microarrays and confirmed using the miRDB database. **Results**: *APH1A*, *CTBP1*, *DTX1*, *HEY1*, *HEY2*, *JAG2*, *NOTCH4*, *TLE2*, and *TLE4* were consistently dysregulated across all breast cancer subtypes. Overexpression of *HEY1* and *JAG2* may be driven by decreased levels of miR-145, miR-98, and miR-381. Conversely, downregulation of *TLE4* may be associated with elevated expression of miR-196a and miR-155. No regulatory miRNAs meeting the selection criteria were identified for *APH1A*, *CTBP1*, *DTX1*, *HEY2*, *NOTCH4*, or *TLE2*. **Conclusions**: The consistent alterations suggest the presence of a shared Notch-driven oncogenic signature in breast cancer, potentially driving cell proliferation, stemness, and resistance to therapy. These findings enhance our understanding of Notch signaling in breast cancer and propose novel miRNA–Notch interactions as candidate targets for therapeutic intervention.

## 1. Introduction

Breast cancer remains the most commonly diagnosed malignancy among women and continues to be a leading contributor to cancer-related mortality on a global scale [1]. Data from the Polish National Cancer Registry indicate that, in 2022, it was the second leading cause of cancer deaths among women, accounting for 23.55% of all such cases. Notably, in women aged 20 to 44, breast cancer represented 27.6% of both cancer incidence and mortality [2].

Breast cancer classification relies on the evaluation of hormone receptor status, including estrogen receptor (ER), progesterone receptor (PR), and human epidermal growth factor receptor 2 (HER2) expression [3]. The most prevalent subtype, luminal A, is typically associated with a favorable prognosis and low aggressiveness. It exhibits positivity for ER and PR but lacks HER2 expression [4]. Luminal B, on the other hand, shows greater proliferative activity and thus is linked to a less favorable outcome; it is ER-positive and may or may not express HER2 [5]. The non-luminal HER2-positive subtype is defined by the overexpression of HER2 in the absence of ER and PR [6]. Finally, triple-negative breast cancer (TNBC), considered the most aggressive variant, is characterized by the lack of ER, PR, and HER2 expression [7].

The Notch signaling pathway is a mechanism of intercellular communication that plays a crucial role in controlling cell fate, proliferation, and differentiation. In mammals, this pathway comprises four Notch receptors (NOTCH1-4) and five canonical ligands: jagged 1 (JAG1), jagged 2 (JAG2), delta-like protein 1 (DLL1), delta-like protein 3 (DLL3), and delta-like protein 4 (DLL4) [8]. Upon ligand binding, the Notch receptor undergoes proteolytic cleavage by γ-secretase, resulting in the release of the Notch intracellular domain, which then translocates to the nucleus to regulate gene expression. This transcriptional activity is modulated by downstream effectors such as hes family bHLH transcription factors (HES) and transcription factors with YRPW motif (HEY). It is also negatively regulated by cofactors, including members of the transducin-like enhancer of split (TLE) family [9,10].

The involvement of the Notch signaling pathway in breast cancer is multifaceted and highly dependent on the tumor context [11]. In some subtypes, such as TNBC, Notch signaling has been shown to exert oncogenic effects, promoting tumor initiation, therapy resistance, and the preservation of cancer stem cell populations [12]. However, other subtypes may exhibit a more nuanced relationship with Notch activity. For instance, overexpression of the intracellular domain of Notch2 has been reported to suppress tumor growth, and elevated levels of Notch2 correlate with better patient survival outcomes [13,14]. The complexity of Notch function stems from its cross-talk with numerous other signaling pathways, such as TGF-β, Wnt/β-catenin, PI3K/AKT, NF-κB, Hippo [11].

In recent years, microRNAs (miRNAs) have been recognized as key post-transcriptional regulators of Notch pathway components. They can suppress translation or promote mRNA degradation, thereby modulating Notch pathway activity [15]. Considering the intricate and context-specific roles of Notch signaling, understanding its regulation by miRNAs may provide deeper insights into the molecular mechanisms underlying breast cancer development and progression, and could aid in the identification of novel therapeutic targets.

The aim of the study was to determine miRNAs that could potentially regulate the expression of genes involved in the Notch signaling pathway across five subtypes of breast cancer in Polish women.

## 2. Materials and Methods

### 2.1. Patients

The study cohort comprised 405 patients. All tissue samples were collected prospectively during primary surgery. Fresh tumor and adjacent non-tumor tissues were rinsed briefly in sterile PBS to remove blood residues and were immediately transferred into a tube containing 1 mL of the TRIzol Reagent (Invitrogen Life Technologies, Carlsbad, CA, USA; cat. no. 15596026) within 30 min of excision and stored at −80 °C. None of the patients received neoadjuvant or adjuvant systemic therapy prior to tissue collection. All cases were classified as T1N0M0 at diagnosis. Following a pathological assessment, 130 neoplastic samples were identified as luminal A, 100 as HER2-negative luminal B, 96 as HER2-positive luminal B, 36 as non-luminal HER2-positive, and 43 as TNBC. Samples of adjacent healthy tissue served as controls. The study was approved by the Bioethical Committee of the Regional Medical Chamber in Kraków (81/KBL/OIL/2023, approval date: 10 March 2023). Informed consent was obtained from all patients.

Detailed patient characteristics are provided in Table 1.

### 2.2. Total RNA Isolation

Total RNA was isolated using TRIzol Reagent (Invitrogen Life Technologies, Carlsbad, CA, USA; cat. no. 15596026). The RNA extracts were subsequently purified with the RNeasy Mini Kit (QIAGEN, Hilden, Germany; cat. no. 74104) and treated with DNase I (Fermentas International Inc., Burlington, ON, Canada; cat. no. 18047019). To evaluate RNA integrity and concentration, samples underwent electrophoresis on a 1% agarose gel, followed by spectrophotometric absorbance measurements. To ensure RNA integrity, all samples were handled on ice, and RNA integrity was confirmed (RIN ≥ 7.0). Samples were stored at −80 °C until analysis.

### 2.3. mRNA Microarrays

Microarray profiling was performed using HG-U133A 2.0 arrays (Affymetrix, Santa Clara, CA, USA) in combination with the GeneChip™ 3′IVT PLUS kit (Thermo Fisher Scientific, Inc., Waltham, MA, USA; cat. no. 902416).

To characterize genes associated with the Notch signaling pathway, we first evaluated pathway activity using the HALLMARK_NOTCH_SIGNALING gene set from the Molecular Signatures Database (MSigDB). For each sample, log_2_-normalized expression values of all available hallmark genes were extracted from the microarray dataset. Gene-wise z-scores were calculated across all samples, and a Notch activity score was computed for each sample as the mean z-score across all hallmark genes. Differences in Notch activity between breast cancer subtypes and control tissue were assessed using the Kruskal–Wallis test followed by Dunn’s post hoc tests with Benjamini–Hochberg correction.

In a subsequent step, to examine the broader Notch-related transcriptomic landscape, genes annotated in the Kyoto Encyclopedia of Genes and Genomes (KEGG) pathway database (entry hsa04330) were utilized [16]. This approach yielded 62 genes, corresponding to 161 mRNA transcripts represented on the microarray platform.

### 2.4. Reverse Transcription Quantitative Polymerase Chain Reaction (RT-qPCR)

To confirm the microarray findings for the KEGG-derived Notch pathway genes, RT-qPCR was conducted using the SensiFast SYBR No-ROX One-Step Kit (Bioline, London, UK). Nine genes that exhibited significant expression changes across all breast cancer subtypes were selected for validation: *APH1A*, *CTBP1*, *DTX1*, *HEY1*, *HEY2*, *JAG2*, *NOTCH4*, *TLE2*, *TLE4*, as listed in Table 2. Gene expression levels were quantified using the 2^−ΔΔCt^ method, with β-actin (*ACTB*) serving as the reference gene.

### 2.5. Enzyme-Linked Immunosorbent Assay (ELISA)

Protein levels were measured using ELISA with commercially available kits targeting the selected proteins. The following kits from MyBioSource, Inc. (San Diego, CA, USA) were used: APH1A (cat. no. MBS760798), CTBP1 (cat. no. MBS7219675), DTX1 (cat. no. MBS720456), HEY2 (cat. no. MBS2905297), JAG2 (cat. no. MBS2020116), NOTCH4 (cat. no. MBS2088207). The kits from Abbexa (Cambridge, UK) were also used: HEY1 (cat. no. abx527055), TLE2 (cat. no. abx549831), TLE4 (cat. no. abx549836).

### 2.6. miRNA Analysis and Prediction

To identify miRNAs distinguishing breast cancer from the control, miRNA Microarray 2.0 (Affymetrix, Santa Clara, CA, USA) was employed, along with the FlashTag Biotin HSR RNA Labeling Kit and the Hybridization Wash and Stain Kit (Affymetrix, Santa Clara, CA, USA). To predict potential regulatory interactions between miRNAs and the selected genes, *APH1A*, *CTBP1*, *DTX1*, *HEY1*, *HEY2*, *JAG2*, *NOTCH4*, *TLE2*, *TLE4*, the mirDB database (http://mirdb.org) was used. A target prediction score threshold of ≥80 was applied to enhance confidence in the identified miRNA-mRNA associations [17].

### 2.7. Statistical Analysis

Microarray data were processed using the Transcriptome Analysis Console (Thermo Fisher Scientific, Waltham, MA, USA). Statistical evaluation was performed via one-way ANOVA followed by Tukey’s post hoc test, applying thresholds of *p* < 0.05 and fold change (FC) > 2 or <−2 for significance. Microarray data were corrected for multiple testing using Benjamini–Hochberg FDR < 0.05.

RT-qPCR and ELISA results were analyzed using Statistica 13.3 (StatSoft, Krakow, Poland). The Shapiro–Wilk test was applied to evaluate data distribution, revealing a non-normal distribution, which justified the use of the Kruskal–Wallis test followed by Dunn’s post hoc test for group comparisons.

Sample size estimation was conducted using G*Power 3.1.9.718 [18]. Based on a one-way ANOVA with *f* = 0.25, α = 0.05, and power = 0.95, the minimum required sample size was calculated to be 324. As the study included 405 patients, a post hoc power analysis indicated a statistical power of 0.99.

Survival data for our cohort were not available due to short follow-up; therefore overall survival (OS) analysis for the different breast cancer subtypes was performed using the Kaplan–Meier plotter (http://kmplot.com/; accessed on 23 June 2025) [19,20], with a follow-up threshold of 60 months. For each gene, patients were stratified into “high” and “low” expression groups using the median cutoff. We also conducted a multigene survival analysis integrating all ten genes simultaneously. Patients were stratified by median signature score (Figure S1).

## 3. Results

### 3.1. Notch Signaling Pathway Activity Analysis Based on MSigDB Hallmark Signature

**Figure 1:**
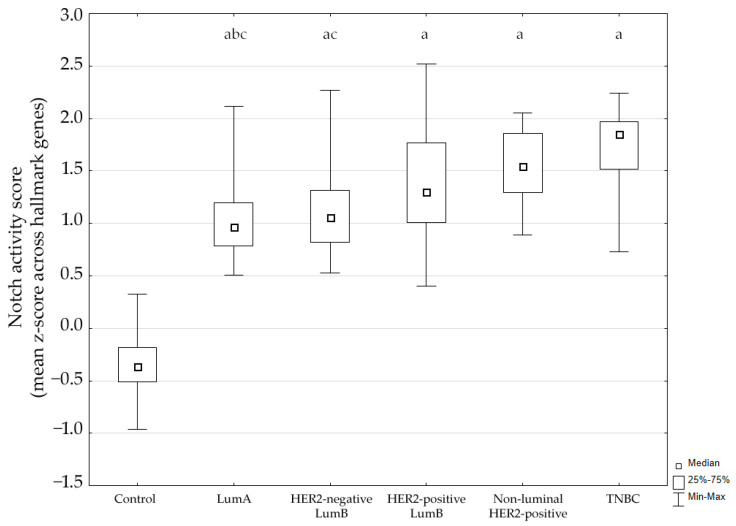
Notch signaling pathway activity across breast cancer subtypes. LumA, luminal A; LumB, luminal B; HER2, human epidermal growth factor receptor 2; TNBC, triple-negative breast cancer. ^a^ *p* < 0.05 vs. Control. ^b^ *p* < 0.05 vs. non-luminal HER2-positive. ^c^ *p* < 0.05 vs. TNBC.

Using the MSigDB HALLMARK_NOTCH_SIGNALING gene set, we calculated a Notch activity score for each sample based on the mean gene-wise z-score across hallmark genes. The score differed significantly between groups (Kruskal–Wallis *p* < 0.001). Post hoc analysis showed significantly higher Notch activity in tumors compared to control breast tissue (*p* < 0.05). Additionally, expression in luminal A cancer was significantly lower compared to the non-luminal HER2-positive subtype and TNBC. The distribution of activity scores across subtypes is shown in Figure 1.

These findings indicate that the Notch pathway is transcriptionally activated across breast cancer subtypes.

### 3.2. mRNA Microarray-Based Gene Expression Profiling

**Figure 2:**
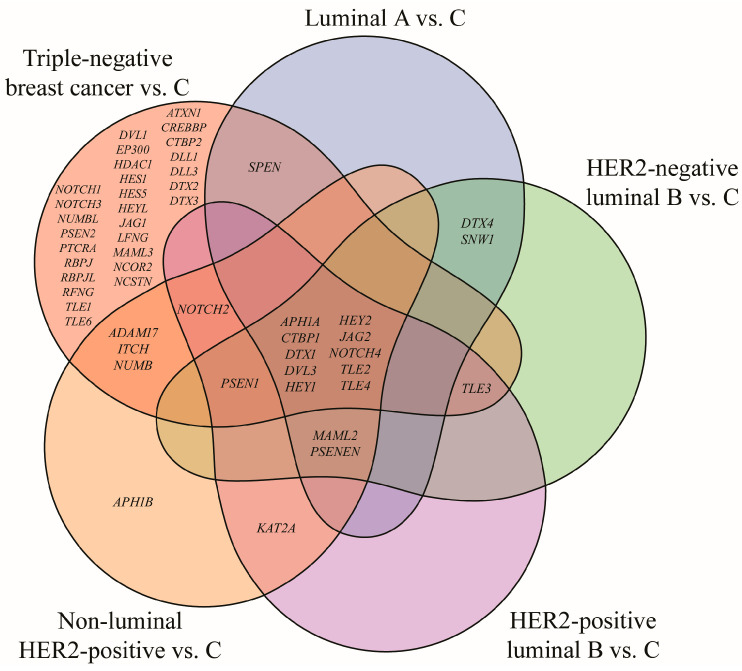
Venn diagram illustrating genes from the Notch signaling pathway that significantly differentiate breast cancer subtypes from the control (*p* < 0.05; FC > 2 or <−2). HER2, human epidermal growth factor receptor 2; C, control; *ADAM17*, ADAM metallopeptidase domain 17; *APH1A*, aph-1A gamma-secretase subunit; *APH1B*, aph-1B gamma-secretase subunit; *ATXN1*, ataxin 1; *CREBBP*, CREB-binding protein; *CTBP1*, C-terminal binding protein 1; *CTBP2*, C-terminal binding protein 2; *DLL1*, delta-like protein 1; *DLL3*, delta-like protein 3; *DTX1*, deltex E3 ubiquitin ligase 1; *DTX2*, deltex E3 ubiquitin ligase 2; *DTX3*, deltex E3 ubiquitin ligase 3; *DTX4*, deltex E3 ubiquitin ligase 4; *DVL1*, disheveled segment polarity protein 1; *DVL3*, disheveled segment polarity protein 3; *EP300*, E1A binding protein p300; *HDAC1*, histone deacetylase 1; *HES1*, hes family bHLH transcription factor 1; *HES5*, hes family bHLH transcription factor 5; *HEY1*, hes-related family bHLH transcription factor with YRPW motif 1; *HEY2*, hes-related family bHLH transcription factor with YRPW motif 2; *HEYL*, hes-related family bHLH transcription factor with YRPW motif-like; *ITCH*, itchy E3 ubiquitin protein ligase; *JAG1*, jagged 1; *JAG2*, jagged 2; *KAT2A*, lysine acetyltransferase 2A; *LFNG*, LFNG O-fucosylpeptide 3-beta-N-acetylglucosaminyltransferase; *MAML2*, mastermind-like transcriptional coactivator 2; *MAML3*, mastermind-like transcriptional coactivator 3; *NCOR2*, nuclear receptor corepressor 2; *NOTCH1*, notch 1; *NOTCH2*, notch 2; *NOTCH3*, notch 3; *NOTCH4*, notch 4; *NUMB*, NUMB endocytic adaptor protein; *NUMBL*, NUMB like endocytic adaptor protein; *PSEN1*, presenilin 1; *PSEN2*, presenilin 2; *PSENEN*, presenilin enhancer gamma secretase subunit; *PTCRA*, pre T-cell antigen receptor alpha; *RBPJ*, recombination signal binding protein for immunoglobulin kappa J region; *RBPJL*, recombination signal binding protein for immunoglobulin kappa J region-like; *RFNG*, RFNG, O-fucosylpeptide 3-beta-N-acetylglucosaminyltransferase; *SNW1*, SNW domain containing 1; *SPEN*, spen family transcriptional repressor; *TLE1*, transducin-like enhancer of split 1; *TLE2*, transducin-like enhancer of split 2; *TLE3*, transducin-like enhancer of split 3; *TLE4*, transducin-like enhancer of split 4; *TLE6*, transducin-like enhancer of split 6.

Out of 161 mRNA transcripts corresponding to 62 genes involved in the Notch signaling pathway (KEGG pathway database, entry hsa04330, [16]), 75 exhibited statistically significant expression changes in breast cancer samples compared to the control (one-way ANOVA, *p* < 0.05; FC > 2 or <−2). Tukey’s post hoc analysis further indicated subtype-specific alterations: 17 mRNAs were differentially expressed in the luminal A subtype, 18 in HER2-negative luminal B subtype, 18 in HER2-positive luminal B subtype, 23 in non-luminal HER2-positive subtype, 69 mRNAs in TNBC. A Venn diagram illustrating both overlapping and distinct genes across subtypes is shown in Figure 2.

Analysis revealed the lack of characteristic genes for luminal A, HER2-negative and HER2-positive luminal B subtypes. Changes in *APH1B* expression were characteristic for the non-luminal HER2-positive subtype, while the highest number of characteristic genes, i.e., 28, was reported for TNBC. Furthermore, ten genes differentiated breast cancer regardless of its subtype: *APH1A*, *CTBP1*, *DTX1*, *DVL3*, *HEY1*, *HEY2*, *JAG2*, *NOTCH4*, *TLE2*, and *TLE4*. *DVL3* has already been described in this cohort in another published work and was therefore omitted from this analysis [21] (Table 3).

Analysis revealed overexpression of *APH1A*, *CTBP1*, *HEY1*, *HEY2*, *JAG2*, *NOTCH4* regardless of the cancer subtype. In addition, *DTX1*, *TLE2*, *TLE4* were significantly downregulated in this study.

### 3.3. Expression Profile of APH1A, CTBP1, DTX1, HEY1, HEY2, JAG2, NOTCH4, TLE2, TLE4 Assessed with RT-qPCR and ELISA

**Figure 3:**
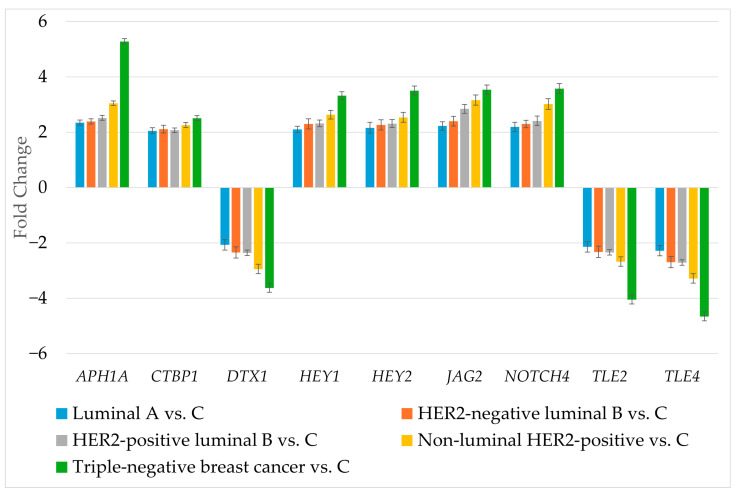
Expression profile of genes differentiating breast cancer regardless of its subtype determined by RT-qPCR. HER2, human epidermal growth factor receptor 2; C, control; *APH1A*, aph-1A gamma-secretase subunit; *CTBP1*, C-terminal binding protein 1; *DTX1*, deltex E3 ubiquitin ligase 1; *HEY1*, hes-related family bHLH transcription factor with YRPW motif 1; *HEY2*, hes-related family bHLH transcription factor with YRPW motif 2; *JAG2*, jagged 2; *NOTCH4*, notch 4; *TLE2*, transducin-like enhancer of split 2; *TLE4*, transducin-like enhancer of split 4. Data are presented as mean ± standard deviation.

RT-qPCR was used to determine the expression of genes differentiating breast cancer regardless of its subtype (Figure 3).

The RT-qPCR results corresponded to the results obtained during microarray experiment. The next step was to determine the protein levels of the selected genes (Table 4).

The level of APH1A, CTBP1, HEY1, HEY2, JAG2, NOTCH4 was significantly higher in cancer samples compared to the control group, while the concentration of DTX1 TLE2, TLE4 was below the detection threshold. This observation is consistent with experiments at the mRNA level.

### 3.4. miRNA Target Prediction

The next step was to verify whether *APH1A*, *CTBP1*, *DTX1*, *HEY1*, *HEY2*, *JAG2*, *NOTCH4*, *TLE2*, and *TLE4* could be targets of miRNAs that differentiate breast cancer from the control (Table 5). The distribution of dysregulated miRNAs across the five breast cancer subtypes is summarized in Supplementary Figure S2, showing the number of unique and shared miRNAs per subtype.

The analysis showed that identified miRNAs did not target *APH1A*, *CTBP1*, *DTX1*, *HEY2*, *NOTCH4*, and *TLE2*. Overexpression of *HEY1* could be linked with low activity of miR-145. Furthermore, there was a relationship between high *JAG2* expression and decreased levels of miR-98 and miR-381. Our analysis also revealed that decreased *TLE4* may be a consequence of increased activity of miR-196a and miR-155.

### 3.5. Overall Survival Outcomes Across Breast Cancer Subtypes

**Figure 4:**
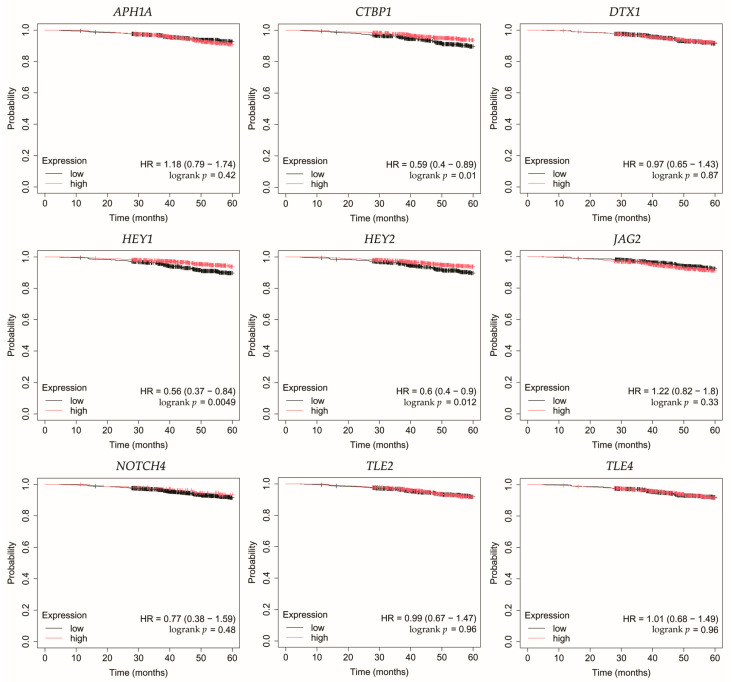
Overall survival analysis for patients with luminal A breast cancer subtype. *APH1A*, aph-1A gamma-secretase subunit; *CTBP1*, C-terminal binding protein 1; *DTX1*, deltex E3 ubiquitin ligase 1; *HEY1*, hes-related family bHLH transcription factor with YRPW motif 1; *HEY2*, hes-related family bHLH transcription factor with YRPW motif 2; *JAG2*, jagged 2; *NOTCH4*, notch 4; *TLE2*, transducin-like enhancer of split 2; *TLE4*, transducin-like enhancer of split 4.

**Figure 5:**
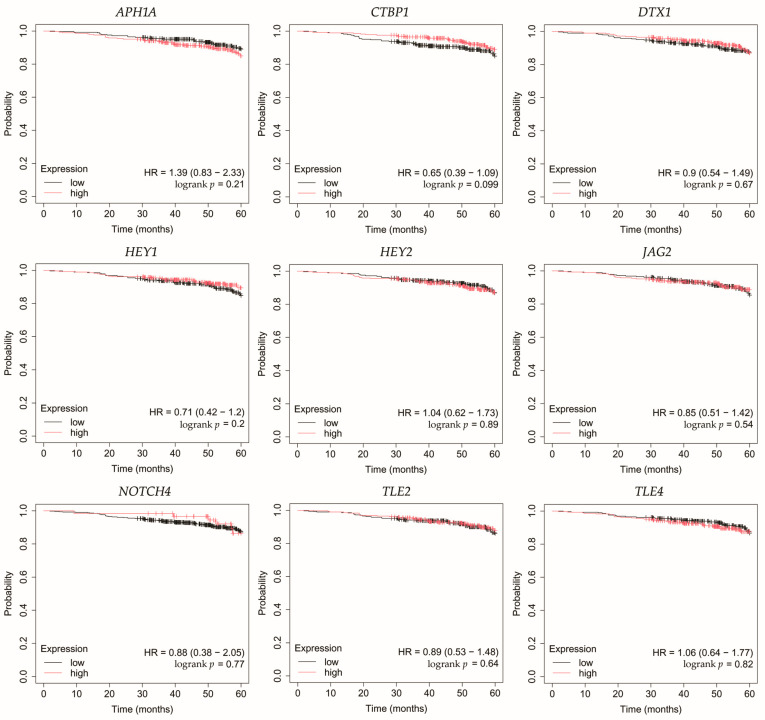
Overall survival analysis of patients with HER2-negative luminal B breast cancer subtype. *APH1A*, aph-1A gamma-secretase subunit; *CTBP1*, C-terminal binding protein 1; *DTX1*, deltex E3 ubiquitin ligase 1; *HEY1*, hes-related family bHLH transcription factor with YRPW motif 1; *HEY2*, hes-related family bHLH transcription factor with YRPW motif 2; *JAG2*, jagged 2; *NOTCH4*, notch 4; *TLE2*, transducin-like enhancer of split 2; *TLE4*, transducin-like enhancer of split 4.

**Figure 6:**
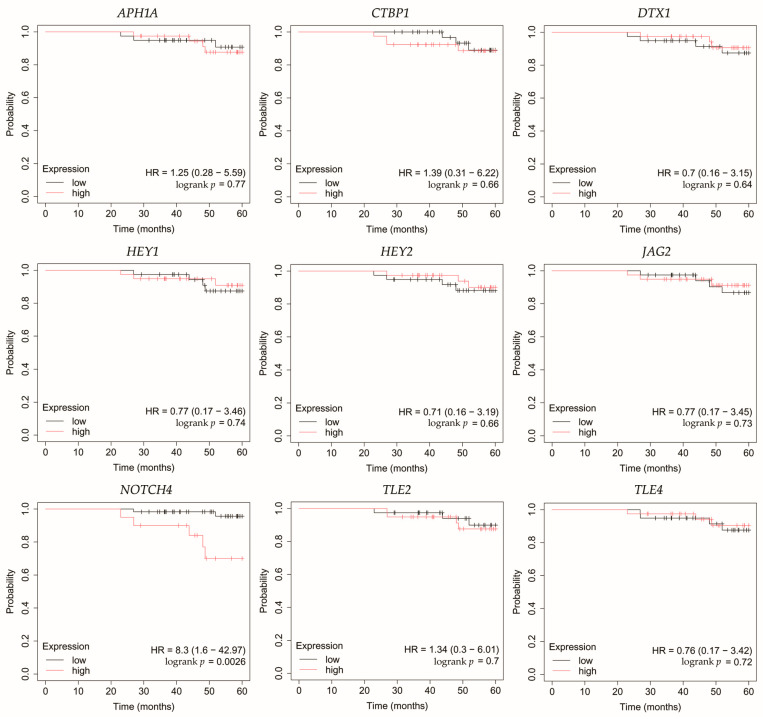
Overall survival analysis of patients with HER2-positive luminal B breast cancer subtype. *APH1A*, aph-1A gamma-secretase subunit; *CTBP1*, C-terminal binding protein 1; *DTX1*, deltex E3 ubiquitin ligase 1; *HEY1*, hes-related family bHLH transcription factor with YRPW motif 1; *HEY2*, hes-related family bHLH transcription factor with YRPW motif 2; *JAG2*, jagged 2; *NOTCH4*, notch 4; *TLE2*, transducin-like enhancer of split 2; *TLE4*, transducin-like enhancer of split 4.

**Figure 7:**
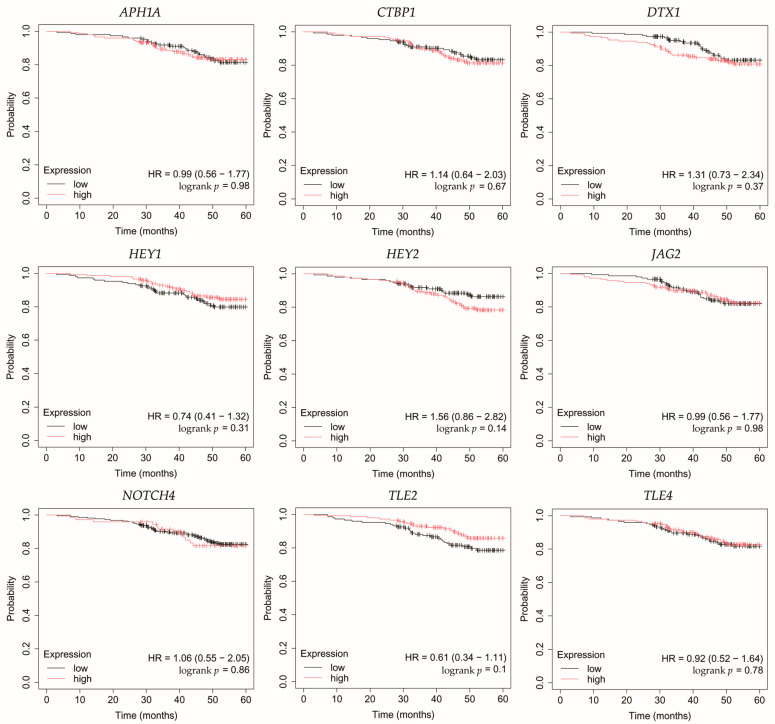
Overall survival analysis of patients with the non-luminal HER2-positive breast cancer subtype. *APH1A*, aph-1A gamma-secretase subunit; *CTBP1*, C-terminal binding protein 1; *DTX1*, deltex E3 ubiquitin ligase 1; *HEY1*, hes-related family bHLH transcription factor with YRPW motif 1; *HEY2*, hes-related family bHLH transcription factor with YRPW motif 2; *JAG2*, jagged 2; *NOTCH4*, notch 4; *TLE2*, transducin-like enhancer of split 2; *TLE4*, transducin-like enhancer of split 4.

**Figure 8:**
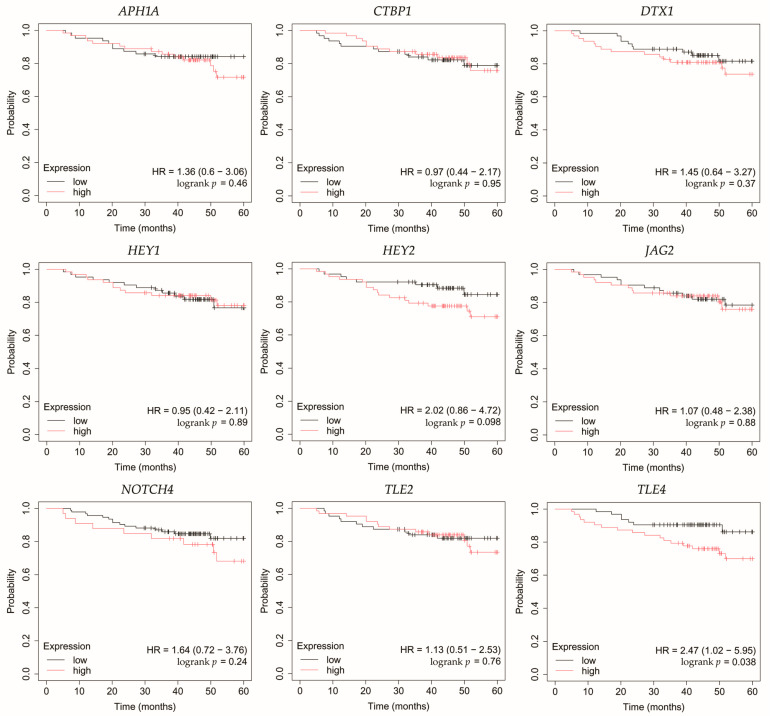
Overall survival analysis of patients with triple-negative breast cancer. *APH1A*, aph-1A gamma-secretase subunit; *CTBP1*, C-terminal binding protein 1; *DTX1*, deltex E3 ubiquitin ligase 1; *HEY1*, hes-related family bHLH transcription factor with YRPW motif 1; *HEY2*, hes-related family bHLH transcription factor with YRPW motif 2; *JAG2*, jagged 2; *NOTCH4*, notch 4; *TLE2*, transducin-like enhancer of split 2; *TLE4*, transducin-like enhancer of split 4.

Overall survival (OS) analysis was conducted for the selected genes: *APH1A*, *CTBP1*, *DTX1*, *HEY1*, *HEY2*, *JAG2*, *NOTCH4*, *TLE2*, and *TLE4* (Figure 4, Figure 5, Figure 6, Figure 7 and Figure 8). Kaplan–Meier survival curves were generated using KMplotter with median cutoff for patient stratification (no optimized thresholds). In addition, a multigene survival analysis, combining all ten common Notch-related genes into a single signature, was performed. The results are shown in Supplementary Figure S1 and did not reach statistical significance.

In luminal A cancer, low expression of *CTBP1*, *HEY1*, *HEY2* was associated with worse OS (Figure 4).

In HER2-negative luminal B cancer, changes in the expression of selected genes was not linked with worse OS (Figure 5).

In HER2-positive luminal B cancer, overexpression of *NOTCH4* was linked with worse OS (Figure 6).

In non-luminal HER2-positive cancer, changes in the expression of selected genes was not linked with worse OS (Figure 7).

In TNBC, high expression of *TLE4* was associated with worse OS (Figure 8).

In addition to evaluating OS for individual Notch pathway components measured in our cohort, we further assessed the prognostic relevance of downstream Notch target genes defined by the MSigDB HALLMARK_NOTCH_SIGNALING signature. Several hallmark genes showed significant associations with overall survival in a subtype-specific manner. In luminal A tumors, low expression of *CCND1*, *DTX2*, *NOTCH1*, *SAP30* and high expression of *KAT2* was linked to poorer outcomes, whereas in HER2-negative luminal B tumors low *TCF7L2* expression was associated with worse OS. In the case of HER2-positive luminal B subtype, changes in the expression of Notch hallmarks genes was not linked to worse outcomes. In non-luminal HER2-positive cancer high *LFNG* expression and low *RBX1* expression was associated with worse OS. Furthermore, low *PRKCA* expression and high *SAP30* expression was linked to poorer outcomes in TNBC. Detailed HR values, confidence intervals and *p*-values for all 32 hallmark genes are provided in Supplementary Table S1.

## 4. Discussion

In our study, we examined the expression patterns of selected components of the Notch pathway across five molecular subtypes of breast cancer: luminal A, HER2-negative luminal B, HER2-positive luminal B, non-luminal HER2-positive, TNBC. We identified consistent upregulation of *APH1A*, *CTBP1*, *HEY1*, *HEY2*, *JAG2*, *NOTCH4* with downregulation of *DTX1*, *TLE2*, *TLE4*, at both the mRNA and protein levels. These changes appeared to be independent of molecular subtype, indicating that core elements of the Notch pathway may play a fundamental role in breast cancer pathogenesis. Subsequent analysis also identified several mRNAs that may regulate expression of these genes.

Among the key findings was the overexpression of *NOTCH4*, a Notch receptor known to promote stemness, invasiveness, and treatment resistance, particularly in aggressive breast cancer subtypes [22,23,24]. Interestingly, Tian et al. found that although Notch4 silencing inhibits metastasis in a TNBC cell line, it also increases tumorigenesis. Therefore, they do not identify Notch4 as a potential therapeutic target in this subtype of breast cancer [25]. On the other hand, Eng et al. demonstrated improved control of breast tumor growth in a murine model after treatment with an anti-Notch4 agent [26]. Additionally, none of the candidate miRNAs meeting our criteria were predicted to regulate its expression.

*JAG2*, a canonical Notch ligand, was also upregulated, suggesting enhanced ligand–receptor interaction and activation of downstream signaling. Moreover, high *JAG2* levels were also associated with poorer OS in TNBC patients. Xing et al. confirmed high levels of JAG2 and Notch signaling in breast cancer, particularly in hypoxia. This was associated with the induction of epithelial-to-mesenchymal transition (EMT) and increased tumor cell survival. Furthermore, they demonstrated that increased *JAG2* expression in bone marrow stroma contributes to the growth of cancer stem-like cells [27]. This was also confirmed by Li et al. in TNBC, where *JAG2* knockdown inhibited resistance to paclitaxel and cancer stemness, which was associated with miR-200 activity [28]. In our study, we observed that *JAG2* overexpression may be associated with low levels of miR-98 and miR-381. Reduced expression of miR-98 in breast cancer has been mostly linked to more aggressive tumor subtypes and unfavorable therapeutic outcomes [29]. Interestingly, there was one study where the level of this miRNA was higher in formalin-fixed paraffin-embedded breast cancer tissues compared to healthy tissues [30]. Modulating miR-98 levels may offer therapeutic benefits by impacting tumor proliferation, invasiveness, and responsiveness to treatment [31,32]. In the case of miR-381, its low activity observed in our study was confirmed by other researchers. Yu et al. indicated that it plays a significant role in the proliferation and invasion of breast cancer cells. Overexpression of miR-381 inhibited EMT by affecting the TGF-β pathway [33]. Furthermore, Shojaei et al. demonstrated the possibility of administering miR-381 mimics to TNBC cells via exosomes isolated from adipose-derived mesenchymal stem cells. As a result, proliferation and invasion were inhibited and apoptosis of breast cancer cells was promoted [34]. Together, the concurrent elevation of *NOTCH4* and *JAG2* supports sustained Notch signaling across diverse tumor subtypes, which in the case of *JAG2* may be additionally related to the lack of inhibitory action by miRNAs.

In line with increased receptor activity, we observed overexpression of the Notch target genes *HEY1* and *HEY2*. Park et al. identified *HEY1* as a predictor of lung metastasis risk in breast cancer patients [35]. Brabletz et al. demonstrated that knockdown of *ZEB1*, an EMT inducer, resulted in decreased activity of the Notch pathway, including *HEY1*. Additionally, *ZEB1* expression was regulated by miR-200 [36]. In our study, we observed that *HEY1* may be a target of miR-145, which showed decreased activity regardless of breast cancer subtype. This is consistent with previous studies indicating tumor suppressor properties of miR-145 [37,38,39,40]. In the case of *HEY2*, Hamelin et al. identified it as a regulator of EMT genes in metastatic breast cancer [41]. Moreover, *HEY2* overexpression is associated with poorer overall survival in basal breast cancer [42]. Simões et al. demonstrated reduced OS in ER^+^ breast cancers and an association with metastasis in the case of high *HEY1* and *HEY2* expression [43]. In our study, high *HEY2* levels were likely not associated with the activity of the identified miRNAs.

*APH1A*, a γ-secretase subunit, was also overexpressed, which may further potentiate Notch receptor cleavage and activation. Yousefi et al. also reported high levels of *APH1A* in breast cancer [44]. Furthermore, overexpression of APH1A resulting from hypoxia in the tumor environment may ultimately promote growth and invasion, but more research is needed [45]. In our study, we also noted elevated levels of *CTBP1*, a transcriptional co-repressor that could facilitate the repression of tumor suppressors. These observations are consistent with previous studies, which found that high levels of CTBP1 may be a potential therapeutic target [46,47]. For both *APH1A* and *CTBP1*, we found no association with the miRNAs identified in our study.

Conversely, we noted reduced expression of *DTX1*, a negative regulator that facilitates ubiquitination and degradation of Notch receptors [48]. Liu et al. also demonstrated that low levels of this gene were associated with the promotion of proliferation, migration, and invasion in breast cancer [49]. Agarwal et al. indicated that *DTX1* downregulation primarily affects early-stage HER2-positive tumors with absent ER and PR [50]. In our study, we also noted that the identified miRNAs did not target *DTX1*.

*TLE2* and *TLE4*, both of which act as transcriptional co-repressors of Notch targets, were downregulated in our study. High levels of *TLE2* were associated with a better prognosis in bladder [51] and pancreatic [52] cancers. However, there is a lack of research on this gene in breast cancer. In our study, we demonstrated reduced *TLE2* expression in all breast cancer subtypes, which was likely unrelated to the activity of the identified miRNAs. In the case of *TLE4*, it is known that its polymorphisms are associated with shorter progression-free survival after gemcitabine treatment in patients with HER2-negative metastatic breast cancer [53]. In our study, *TLE4* reduced expression may be related to the overexpression of miR-196a and miR-155. Previous studies have identified miR-196a as an oncogene in breast cancer and a potential therapeutic target [54,55,56]. Xiong et al. also demonstrated that it has diagnostic value and that its overexpression is associated with poor survival [57]. Furthermore, miR-155 is also considered a promising biomarker for breast cancer [58]. Wang et al. reported its suppressor role in breast cancer, where its excessive activity recruits immune cells [59]. On the other hand, other studies emphasize its usefulness in potential therapy aimed at inhibiting miR-155 activity, which will result in inhibition of proliferation and increased apoptosis [60,61,62]. Moreover, Chernyy et al. noted that in patients with lymph node positive status who received preoperative neoadjuvant chemotherapy, miR-155 levels were lower than in the untreated group [63].

Although the core Notch pathway components were consistently dysregulated across all breast cancer subtypes, some patterns in TNBC suggest subtype-specific roles. In particular, overexpression of NOTCH1, a receptor frequently activated in TNBC, may contribute to enhanced EMT, tumor invasiveness, and maintenance of cancer stem-like cells [64,65,66]. Similarly, elevated levels of JAG2 and NOTCH4 in TNBC could further sustain Notch signaling, promoting aggressive tumor behavior and therapy resistance [22,23,27,28]. Downregulation of negative regulators such as DTX1 may amplify these effects of Notch signaling [49]. Moreover, dysregulated Notch signaling in TNBC may influence the tumor microenvironment, including immune cell infiltration and stromal interactions, although these aspects require further investigation [67,68]. These observations suggest that even genes commonly dysregulated across subtypes can have amplified functional consequences in TNBC, highlighting the importance of considering subtype-specific context in future mechanistic and therapeutic studies.

Taken together, our findings reveal consistent alterations in the Notch signaling pathway across all molecular subtypes of breast cancer in Polish women. Key components, including *NOTCH4*, *JAG2*, *HEY1*, and *HEY2*, were upregulated regardless of subtype, alongside downregulation of *DTX1*, *TLE2*, and *TLE4*. These patterns suggest a core Notch-driven oncogenic mechanism independent of subtype, contributing to tumor progression, stemness, and therapy resistance. miRNA dysregulation, particularly involving miR-145, miR-98, miR-381, miR-155, and miR-196a, may underpin these expression changes, offering mechanistic insight and potential therapeutic targets. Limitations of our study include unequal representation of less common subtypes (non-luminal HER2-positive and TNBC) and restriction to a Polish population, which may affect generalizability. Another important limitation concerns the nature of bulk mRNA profiling. RNA isolated from tumor tissue reflects a composite signal originating not only from malignant epithelial cells but also from stromal components such as immune infiltrates, endothelial cells, fibroblasts, and other mesenchymal cells. As a result, the expression levels of Notch pathway–related genes observed in our dataset cannot be attributed exclusively to cancer cells. This is particularly relevant for genes that are known to be expressed by both tumor and stromal populations. Therefore, some of the dysregulation detected in our cohort may in part reflect microenvironmental contributions rather than purely tumor-intrinsic alterations. In addition, the study focused on prediction-based screening of miRNAs and experimental validation (luciferase, mimics/inhibitors) is a priority for future work. A limitation of our protein analysis is that ELISA performed on tissue homogenates does not distinguish intracellular or membrane-bound fractions from secreted forms of Notch-related proteins. Future work may include assessment of soluble or circulating Notch-related proteins in serum/plasma to determine whether they correlate with transcriptome-derived Notch pathway activity.

This study comprehensively characterized the expression of Notch pathway-related genes and their potential miRNA regulators across five molecular breast cancer subtypes. *APH1A*, *CTBP1*, *DTX1*, *HEY1*, *HEY2*, *JAG2*, *NOTCH4*, *TLE2*, and *TLE4* were consistently dysregulated across all subtypes. Further analysis indicated that miRNAs such as miR-145, miR-98, miR-381, miR-155, and miR-196a may influence these expression patterns.

## 5. Conclusions

The consistent alterations suggest the presence of a shared Notch-driven oncogenic signature in breast cancer, potentially driving cell proliferation, stemness, and resistance to therapy. These findings enhance our understanding of Notch signaling in breast cancer and propose novel miRNA–Notch interactions as candidate targets for therapeutic intervention.

## Figures and Tables

**Table 1:** Summary of patient data included in the study.

Subtype	Grade			Age		BMI [kg/m^2^]
	G1	G2	G3	<50 Years	>50 Years	
Luminal A	23 (18%)	48 (37%)	59 (45%)	43 (33%)	87 (67%)	30.78 ± 2.76
HER2-negative luminal B	31 (31%)	57 (57%)	12 (12%)	32 (32%)	68 (68%)	30.18 ± 4.56
HER2-positive luminal B	23 (24%)	57 (59%)	16 (17%)	19 (20%)	77 (80%)	32.09 ± 6.19
Non-luminal HER2-positive	9 (25%)	12 (33%)	15 (42%)	9 (25%)	27 (75%)	33.18 ± 5.67
TNBC	14 (32%)	21 (49%)	8 (19%)	10 (23%)	33 (77%)	34.67 ± 2.98

HER2, human epidermal growth factor receptor 2; TNBC, triple-negative breast cancer; BMI, body mass index.

**Table 2:** RT-qPCR primer details.

mRNA	RT-qPCR Primers (5′–3′)
*APH1A*	Forward: CTACAGGAGGTGTTCCGCTTTG Reverse: GACACCACTGATGATACCGAAGG
*CTBP1*	Forward: AGATGCCCATCCTGAAGGACGT Reverse: GAGGGCTTTGAACTTCTCCAGG
*DTX1*	Forward: AGAATCCCGAGGATGTGGTTCG Reverse: TCGTAGCCTGATGCTGTGACCA
*HEY1*	Forward: TGTCTGAGCTGAGAAGGCTGGT Reverse: TTCAGGTGATCCACGGTCATCTG
*HEY2*	Forward: TGAGAAGACTTGTGCCAACTGCT Reverse: CCCTGTTGCCTGAAGCATCTTC
*JAG2*	Forward: GCTGCTACGACCTGGTCAATGA Reverse: AGGTGTAGGCATCGCACTGGAA
*NOTCH4*	Forward: TTCCACTGTCCTCCTGCCAGAA Reverse: TGGCACAGGCTGCCTTGGAATC
*TLE2*	Forward: CTGCCTCCAAATCCTGTGACTC Reverse: TGGTGAAGGGACTGGACAGAGT
*TLE4*	Forward: GGAAAACCACCAGGAGTTGACC Reverse: TGGTCAGCTCTCCGTTCATTCC
*ACTB*	Forward: TCACCCACACTGTGCCCATCTACGA Reverse: CAGCGGAACCGCTCATTGCCAATGG

*APH1A*, aph-1A gamma-secretase subunit; *CTBP1*, C-terminal binding protein 1; *DTX1*, deltex E3 ubiquitin ligase 1; *HEY1*, hes-related family bHLH transcription factor with YRPW motif 1; *HEY2*, hes-related family bHLH transcription factor with YRPW motif 2; *JAG2*, jagged 2; *NOTCH4*, notch 4; *TLE2*, transducin-like enhancer of split 2; *TLE4*, transducin-like enhancer of split 4; *ACTB*, β-actin.

**Table 3:** Details of mRNAs representing Notch signaling pathway differentiating breast cancer from the control regardless of its subtype (*p* < 0.05; FC > 2 or <−2).

ID	mRNA	Fold Change				
		LumA vs. C	HER2-Negative LumB vs. C	HER2-Positive LumB vs. C	Non-Luminal HER2-Positive vs. C	TNBC vs. C
1554417_s_at	*APH1A*	2.25	2.23	2.44	3.33	9.31
203392_s_at	*CTBP1*	2.14	2.15	2.24	2.36	2.83
227336_at	*DTX1*	−2.16	−2.34	−2.39	−3.15	−4.01
218839_at 44783_s_at	*HEY1*	2.02 2.96	2.19 2.98	2.22 3.34	2.78 3.43	3.22 3.76
219743_at	*HEY2*	2.05	2.16	2.21	2.8	3.97
209784_s_at 32137_at	*JAG2*	2.28 2.19	2.46 2.63	2.39 2.82	2.53 2.96	2.71 3.85
205247_at	*NOTCH4*	2.36	2.59	2.68	3.24	3.8
40837_at	*TLE2*	−2.09	−2.39	−2.38	−3.01	−5.44
204872_at	*TLE4*	−2.35	−2.46	−3.53	−3.81	−5.18

ID, number of the probe; LumA, luminal A; LumB, luminal B; HER2, human epidermal growth factor receptor 2; TNBC, triple-negative breast cancer; C, control; *APH1A*, aph-1A gamma-secretase subunit; *CTBP1*, C-terminal binding protein 1; *DTX1*, deltex E3 ubiquitin ligase 1; *HEY1*, hes-related family bHLH transcription factor with YRPW motif 1; *HEY2*, hes-related family bHLH transcription factor with YRPW motif 2; *JAG2*, jagged 2; *NOTCH4*, notch 4; *TLE2*, transducin-like enhancer of split 2; *TLE4*, transducin-like enhancer of split 4.

**Table 4:** Concentration of APH1A, CTBP1, DTX1, HEY1, HEY2, JAG2, NOTCH4, TLE2, TLE4 in breast cancer subtypes and control group (*p* < 0.05).

Protein [ng/mL]	Control	LumA	HER2-Negative LumB	HER2-Positive LumB	HER2-Positive	TNBC
APH1A	8.26 ± 0.31	18.34 ± 0.19 *	19.95 ± 0.27 *	20.93 ± 0.27 *	20.53 ± 0.34 *	26.79 ± 0.31 *
CTBP1	2.42 ± 0.19	5.63 ± 0.17 *	5.7 ± 0.26 *	6.08 ± 0.21 *	6.09 ± 0.33 *	6.36 ± 0.23 *
DTX1	1.55 ± 0.2	below detection threshold *	below detection threshold *	below detection threshold *	below detection threshold *	below detection threshold *
HEY1	0.95 ± 0.13	2.49 ± 0.22 *	3.1 ± 0.19 *	3.42 ± 0.24 *	3.53 ± 0.24 *	5.5 ± 0.19 *
HEY2	0.71 ± 0.19	2.61 ± 0.14 *	3.96 ± 0.25 *	3.97 ± 0.19 *	5.6 ± 0.34 *	5.78 ± 0.23 *
JAG2	1.8 ± 0.18	2.87 ± 0.13 *	4 ± 0.16 *	4.36 ± 0.19 *	7.05 ± 0.16 *	10.83 ± 0.21 *
NOTCH4	1.63 ± 0.18	2.88 ± 0.13 *	3.18 ± 0.16 *	3.62 ± 0.19 *	4.01 ± 0.15 *	6.15 ± 0.21 *
TLE2	0.78 ± 0.2	below detection threshold *	below detection threshold *	below detection threshold *	below detection threshold *	below detection threshold *
TLE4	2.32 ± 0.2	below detection threshold *	below detection threshold *	below detection threshold *	below detection threshold *	below detection threshold *

LumA, luminal A; LumB, luminal B; HER2, human epidermal growth factor receptor 2; TNBC, triple-negative breast cancer; C, control; APH1A, aph-1A gamma-secretase subunit; CTBP1, C-terminal binding protein 1; DTX1, deltex E3 ubiquitin ligase 1; HEY1, hes-related family bHLH transcription factor with YRPW motif 1; HEY2, hes-related family bHLH transcription factor with YRPW motif 2; JAG2, jagged 2; NOTCH4, notch 4; TLE2, transducin-like enhancer of split 2; TLE4, transducin-like enhancer of split 4. * *p* < 0.05 vs. control.

**Table 5:** Expression of miRNAs potentially involved in the regulation of studied genes (*p* < 0.05; FC > 2 or <−2).

mRNA	miRNA	Target Score	Fold Change				
			LumA vs. C	HER2-Negative LumB vs. C	HER2-Positive LumB vs. C	HER2-Positive vs. C	TNBC vs. C
*HEY1*	miR-145	90	−2.05	−2.14	−2.11	−3.22	−3.77
*JAG2*	miR-98 miR-381	86 86	−2.12 −2.05	−2.16 −2.1	−2.35 −2.43	−2.55 −2.6	−2.83 −2.52
*TLE4*	miR-196a miR-155	100 93	2.06 2.1	2.31 3.21	2.7 3.45	2.76 4.97	3.43 7.84

LumA, luminal A; LumB, luminal B; HER2, human epidermal growth factor receptor 2; TNBC, triple-negative breast cancer; C, control; *HEY1*, hes-related family bHLH transcription factor with YRPW motif 1; *JAG2*, jagged 2; *TLE4*, transducin-like enhancer of split 4.

## Data Availability

All data generated or analyzed during this study are included in this published article.

## Supplementary Materials

The following supporting information can be downloaded at https://www.mdpi.com/article/10.3390/biomedicines13123065/s1, Figure S1: Kaplan–Meier survival curves for the 10-gene Notch signature across breast cancer subtypes. The 10-gene signature was constructed using the mean expression of all selected genes (equal weights, median cutoff); Figure S2: The distribution of dysregulated miRNAs across the breast cancer subtypes; Table S1: Detailed overall survival analysis for Notch hallmark genes.

## Abbreviations

The following abbreviations are used in this manuscript:

APH1A	aph-1A gamma-secretase subunit
CTBP1	C-terminal binding protein 1
DTX1	deltex E3 ubiquitin ligase 1
HER2	human epidermal growth factor receptor 2
HEY1	hes-related family bHLH transcription factor with YRPW motif 1
HEY2	hes-related family bHLH transcription factor with YRPW motif 2
JAG2	jagged 2
miRNA	microRNA
NOTCH4	notch 4
OS	overall survival
TLE2	transducin-like enhancer of split 2
TLE4	transducin-like enhancer of split 4
TNBC	triple-negative breast cancer
