# Supplementary material for: Subtype-Independent Dysregulation of the Notch Signaling Pathway and Its miRNA Regulators in Breast Cancer
Source: Biomedicines. 2025 Dec 12;13(12):3065. doi: 10.3390/biomedicines13123065 (PMC12730416; doi:10.3390/biomedicines13123065)
Supplement: Supplementary file 1 [file biomedicines-13-03065-s001.zip › Table S1.pdf]

biomedicines

MDPI

Table S1. Detailed overall survival analysis for Notch hallmark genes.

Gene	LumA		Direc-tion	HER2-negative LumB		Direction	HER2-positive LumB		Direction	Non-luminal HER2-positive		Direction	TNBC		Direction
	HR	*p*		HR	*p*		HR	*p*		HR	*p*		HR	*p*	
APH1A	1.18 (0.79-1.174)	0.42	NS	1.39 (0.83-2.33)	0.21	NS	1.25 (0.28-5.59)	0.77	NS	0.99 (0.56-1.77)	0.98	NS	1.36 (0.6-3.06)	0.46	NS
ARRB1	1.39 (0.93-2.07)	0.1	NS	1.39 (0.83-2.34)	0.21	NS	2.63 (0.51-13.55)	0.23	NS	0.9 (0.5-1.6)	0.72	NS	1.01 (0.45-2.24)	0.99	NS
CCND1	0.48 (0.31-0.72)	< 0.001	Low worse	0.82 (0.49-1.36)	0.44	NS	0.74 (0.17-3.32)	0.7	NS	0.6 (0.33-1.09)	0.09	NS	0.81 (0.36-1.81)	0.61	NS
CUL1	1.02 (0.69-1.51)	0.93	NS	1.25 (0.75-2.1)	0.39	NS	0.79 (0.18-3.52)	0.76	NS	1.23 (0.69-2.2)	0.48	NS	1.89 (0.82-4.32)	0.13	NS
DLL1	0.69 (0.46-1.02)	0.06	NS	0.86 (0.51-1.43)	0.56	NS	1.38 (0.31-6.17)	0.67	NS	1.63 (.09-2.95)	0.1	NS	1.22 (0.55-2.73)	0.62	NS
DTX1	0.97 (0.65-1.43)	0.87	NS	0.9 (0.54-1.49)	0.67	NS	0.7 (0.16-3.15)	0.64	NS	1.31 (0.73-2.34)	0.37	NS	1.45 (0.64-3.27)	0.37	NS
DTX2	0.61 (0.41-0.91)	0.01	Low worse	0.87 (0.52-1.46)	0.6	NS	0.41 (0.08-2.11)	0.27	NS	0.75 (0.42-1.34)	0.33	NS	1.27 (0.56-2.82)	0.57	NS
DTX4	1.07 (0.72-1.59)	0.73	NS	0.78 (0.47-1.3)	0.34	NS	2.61 (0.513.46)	0.23	NS	0.87 (0.49-1.55)	0.64	NS	0.87 (0.39-1.94)	0.73	NS

FBXW11	1.08 (0.73-1.61)	0.7	NS	1.16 (0.69-1.96)	0.56	NS	0 (0-Inf)	0.12	NS	1.3 (0.72-2.33)	0.38	NS	1.03 (0.43-2.5)	0.94	NS
FZD1	0.96 (0,65-1.42)	0.83	NS	1.2 (0.72-2.01)	0.48	NS	2.52 (0.49-13.06)	0.25	NS	1.08 (0.6-1.92)	0.8	NS	0.8 (0.36-1.78)	0.58	NS
FZD5	0.79 (0.53-1.17)	0.24	NS	0.91 (0.55-1.52)	0.71	NS	1.37 (0.31-6.13)	0.68	NS	1.09 (0.61-1.95)	0.76	NS	1.49 (0.66-3.35)	0.33	NS
FZD7	0.79 (0.54-1.18)	0.25	NS	1.23 (0.74-2.06)	0.42	NS	5.62 (0.68-46.71)	0.07	NS	1.07 (0.6-1.91)	0.82	NS	0.98 (0.44-2.19)	0.97	NS
HES1	0.98 (0.66-1.45)	0.93	NS	0.73 (0.44-1.23)	0.23	NS	0.37 (0.07-1.9)	0.21	NS	1.1 (0.62-1.96)	0.75	NS	1.61 (0.7-3.68)	0.26	NS
HEYL	0.86 (0.58-1.27)	0.44	NS	1.19 (0.72-1.99)	0.5	NS	6.11 (0.74-50.8)	0.06	NS	1.3 (0.73-2.34)	0.37	NS	0.63 (0.28-1.43)	0.27	NS
JAG1	0.89 (0.6-1.31)	0.55	NS	1.09 (0.65-1.81)	0.75	NS	1.38 (0.31-6.19)	0.67	NS	1.24 (0.69-2.23)	0.46	NS	0.96 (0.43-2.14)	0.92	NS
KAT2A	1.53 (1.03-229)	0.04	High worse	1.59 (0.94-2.68)	0.08	NS	1.3 (0.29-5.8)	0.73	NS	0.64 (0.35-1.16)	0.14	NS	0.69 (0.3-1.54)	0.36	NS
LFNG	0.92 (0.62-1.36)	0.68	NS	1.35 (0.8-2.26)	0.25	NS	6.27 (0.75-52-08)	0.05	NS	2.13 (1.15-3.95)	0.01	High worse	1.82 (0.79-4.15)	0.15	NS
MAML2	0.71 (0.48-1.05)	0.09	NS	1.13 (0.67-1.88)	0.65	NS	1.19 (0.27-5.32)	0.82	NS	1.51 (0.84-2.72)	0.16	NS	1.42 (0.63-3.21)	0.39	NS
NOTCH1	0.47 (0.3-0.71)	< 0.001	Low worse	0.86 (0.51-1.44)	0.56	NS	0.39 (0.08-2.01)	0.24	NS	1.1 (0.62-1.96)	0.75	NS	1.06 (0.47-2.36)	0.89	NS
NOTCH2	0.69 (0.47-1.03)	0.07	NS	0.96 (0.58-1.6)	0.88	NS	5.7 (0.69-47.33)	0.07	NS	1.03 (0.53-1.83)	0.93	NS	1.41 (0.62-3.17)	0.41	NS

NOTCH3	0.83 (0.56-1.22)	0.34	NS	1.37 (0.82-2.3)	0.23	NS	0.41 (0.08-2.12)	0.27	NS	1.49 (0.83-2.68)	0.18	NS	1.15 (0.52-2.58)	0.73	NS
PPARD	0.74 (0.5-1.1)	0.13	NS	1.14 (0.68-1.9)	0.62	NS	0.72 (0.16-3.22)	0.67	NS	1.02 (0.57-1.82)	0.94	NS	0.72 (0.32-1.62)	0.43	NS
PRKCA	1.04 (0.71-1.55)	0.83	NS	1.03 (0.62-1.72)	0.91	NS	0.16 (0.02-1.35)	0.06	NS	0.67 (0.37-1.22)	0.19	NS	0.24 (0.09-0.63)	0.002	Low worse
PSEN2	1.05 (0.71-1.55)	0.82	NS	0.91 (0.55-1.52)	0.72	NS	1.32 (0.3-5.92)	0.71	NS	0.96 (0.54-1.72)	0.9	NS	1.83 (0.8-4.19)	0.15	NS
PSENEN	0.99 (0.5-1.97)	0.99	NS	0.62 (0.19-1.97)	0.41	NS	0 (0-Inf)	0.3	NS	0.74 (0.27-2.07)	0.57	NS	1.37 (0.32-5.83)	0.67	NS
RBX1	0.94 (0.63-1.39)	0.74	NS	0.72 (0.43-1.21)	0.22	NS	0.42 (0.08-2.19)	0.29	NS	0.4 (0.21-0.76)	0.004	Low worse	0.92 (0.41-2.06)	0.84	NS
SAP30	0.66 (0.44-0.98)	0.04	Low worse	0.82 (0.49-1.37)	0.44	NS	0.79 (0.18-3.55)	0.76	NS	0.99 (0.55-1.76)	0.96	NS	2.39 (1.02-5.59)	0.04	High worse
SKP1	0.82 (0.55-1.21)	0.32	NS	0.78 (0.47-1.31)	0.35	NS	0.4 (0.08-2.08)	0.26	NS	0.85 (0.48-1.52)	0.59	NS	1.4 (0.62-3.14)	0.42	NS
ST3GAL6	1.29 (0.87-1.91)	0.21	NS	1.1 (0.66-1.83)	0.72	NS	0.72 (0.16-3.2)	0.66	NS	1.59 (0.88-2.86)	0.12	NS	1.17 (0.52-2.61)	0.7	NS
TCF7L2	1.07 (0.72-1.58)	0.75	NS	0.58 (0.34-0.98)	0.04	Low worse	0.39 (0.08-2.02)	0.25	NS	0.73 (0.4-1.3)	0.28	NS	1.5 (0.66-3.37)	0.33	NS
WNT2	0.83 (0.56-1.22)	0.34	NS	0.69 (0.41-1.16)	0.16	NS	1.2 (0.27-5.36)	0.81	NS	0.91 (0.51-1.62)	0.74	NS	1.66 (0.72-3.79)	0.23	NS
WNT5A	0.7 (0.47-1.04)	0.07	NS	0.88 (0.53-1.47)	0.62	NS	1.25 (0.28-5.6)	0.77	NS	0.99 (0.56-1.77)	0.98	NS	1.22 (0.55-2.72)	0.63	NS

LumA, luminal A; LumB, luminal B; HER2, human epidermal growth factor receptor 2; TNBC, triple-negative breast cancer; *APH1A*, aph-1A gamma-secretase subunit; *ARRB1*, arrestin beta 1; *CCND1*, cyclin D1; *CUL1*, cullin 1; *DLL1*, delta like canonical Notch ligand 1; *DTX1*, deltex E3 ubiquitin ligase

1; *DTX2*, deltex E3 ubiquitin ligase 2; *DTX4*, deltex E3 ubiquitin ligase 4; *FBXW11*, F-box and WD repeat domain containing 11; *FZD1*, frizzled class receptor 1; *FZD5*, frizzled class receptor 5; *FZD7*, frizzled class receptor 7; *HES1*, hes family bHLH transcription factor 1; *HEYL*, hes related family bHLH transcription factor with YRPW motif like; *JAG1*, jagged canonical Notch ligand 1; *KAT2A*, lysine acetyltransferase 2A; *LFNG*, LFNG O-fucosylpeptide 3-beta-N-acetylglucosaminyltransferase; *MAML2*, mastermind like transcriptional coactivator 2; *NOTCH1*, notch receptor 1; *NOTCH2*, notch receptor 2; *NOTCH3*, notch receptor 3; *PPARD*, peroxisome proliferator activated receptor delta; *PRKCA*, protein kinase C alpha; *PSEN2*, presenilin 2; *PSENEN*, presenilin enhancer, gamma-secretase subunit; *RBX1*, ring-box 1; *SAP30*, Sin3A associated protein 30; *SKP1*, S-phase kinase associated protein 1; *ST3GAL6*, ST3 beta-galactoside alpha-2,3-sialyltransferase 6; *TCF7L2*, transcription factor 7 like 2; *WNT2*, Wnt family member 2; *WNT5A*, Wnt family member 5A.
