# Supplementary figures and images for: Subtype-Independent Dysregulation of the Notch Signaling Pathway and Its miRNA Regulators in Breast Cancer
Source: Biomedicines. 2025 Dec 12;13(12):3065. doi: 10.3390/biomedicines13123065 (PMC12730416; doi:10.3390/biomedicines13123065)

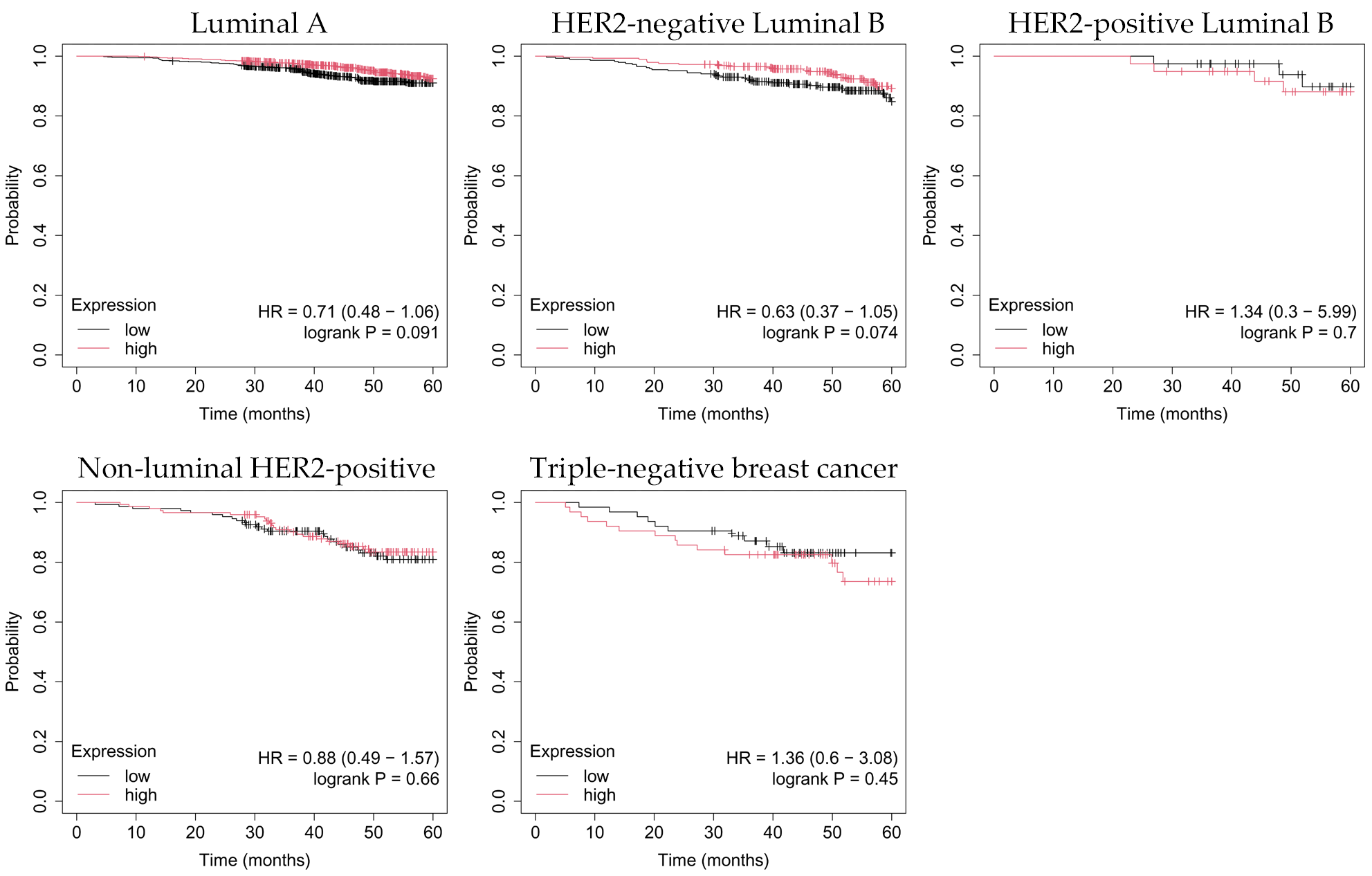

Supplement: Supplementary file 1 [file biomedicines-13-03065-s001.zip › Figure S1.tiff]

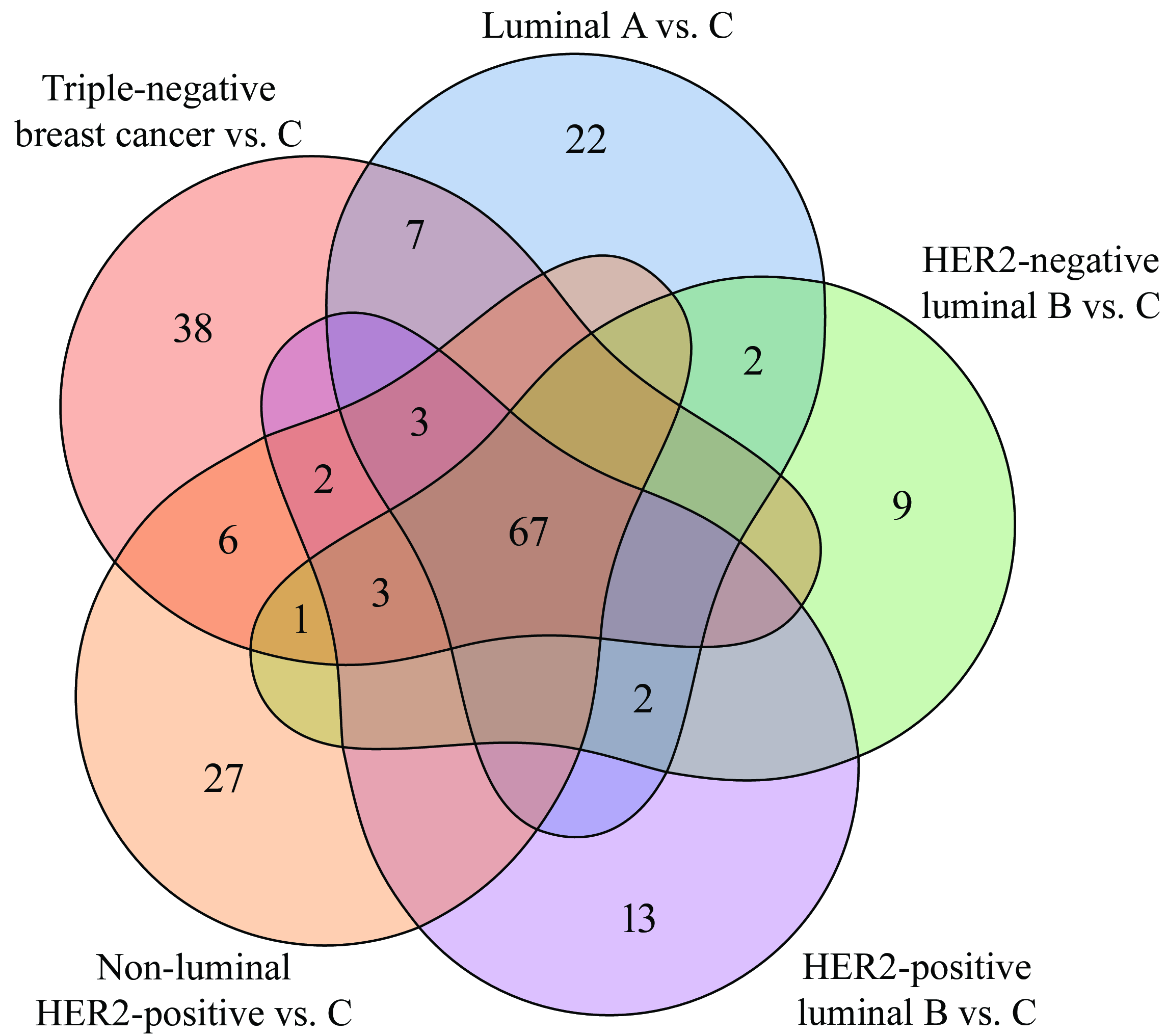

Supplement: Supplementary file 1 [file biomedicines-13-03065-s001.zip › Figure S2.tif]
